# Temporal Predictions in Music and Language: The Case of Autism Spectrum Disorder

**DOI:** 10.1111/nyas.70084

**Published:** 2025-09-15

**Authors:** Maude Denis, Chiara Mazzocconi, David Da Fonseca, Daniele Schön

**Affiliations:** ^1^ Inserm, INS, Institut de Neurosciences des Systèmes Aix Marseille University Marseille France; ^2^ CNRS, CRPN Aix Marseille University Marseille France; ^3^ Service de Pédopsychiatrie AP‐HM, Hôpital Salvator Marseille France

**Keywords:** autism spectrum disorder, conversational alignment, predictive coding, rhythmic synchronization

## Abstract

The predictive coding theory of autism suggests that individuals with autism may show atypicalities in how predictions are formed or updated. This may in turn affect how they process temporal information. While predictive coding has been widely applied to the study of language, including conversation, and music, including musical interactions, relatively few studies have explored the intersection of these domains in autism. Even fewer have focused on the role of temporal predictions in both language and music. This review examines studies that investigate temporal processing and predictive mechanisms in both music and language in individuals with autism spectrum disorder (ASD). Understanding these shared temporal mechanisms is crucial for providing a more comprehensive view of the underlying cognitive processes and difficulties in ASD. Furthermore, exploring the relationship between music and language from a temporal prediction perspective offers valuable insights into more ecologically valid and interactive settings, such as conversation and music‐making. Such research not only improves our understanding of autism but also has important implications for therapeutic interventions, particularly those leveraging rhythmic training to enhance social communication and coordination skills.

## Graphical abstract

This review explores how individuals with autism spectrum disorder (ASD) make predictions and interact in both music and language. By highlighting shared temporal mechanisms and synchronization phenomena across domains, we aim to contribute to a better characterization of coordinative dynamics in ASD. Surprisingly, few studies have directly linked music and speech under this perspective, especially in ASD and ecologically valid interactive settings.

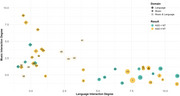

## 1 Theoretical Framework of Autism Spectrum Disorder

Autism spectrum disorder (ASD) is a neurodevelopmental condition defined by persistent difficulties in social communication, alongside restricted, repetitive patterns of behavior, interests, or activities, as outlined in the DSM‐5. With a prevalence at around 1% of the general population, ASD manifests in early developmental periods and significantly impacts everyday functioning.

Beyond the diagnostic criteria, comorbidities are frequently observed in individuals with ASD. These include motor coordination difficulties, attentional challenges, sensory processing anomalies, and anxiety disorders [1, 2, 3, 4, 5, 6, 7]. Such features underline the heterogeneity and complexity of the autistic phenotype.

While ASD encompasses a broad constellation of symptoms, difficulties in interpersonal relationships often stand out as a source of significant distress. Individuals with ASD frequently report experiences of social isolation, misunderstanding, and bullying, contrasting with a rich inner world and a deep desire for connection, summarized poignantly in the words of many: “I just wish I could have a few friends, have a normal life.” Understanding the roots of this social suffering remains a key challenge for both clinical practice and theoretical models of autism.

Several theories have been proposed, focusing primarily on social and communication difficulties. The Theory of Mind deficit [8] suggests difficulties in attributing (predicting) mental states to others, while the Social Motivation Theory [9] emphasizes a reduced drive to engage in social interactions.

Other theories highlight nonsocial factors as the source of the social challenges. The Executive Function Deficit Theory [10] highlights difficulties with planning and cognitive flexibility. The Weak Central Coherence Theory [11] describes a tendency to focus on details over global context. The Enhanced Perceptual Functioning Theory [12] proposes superior low‐level perceptual processing in autism, suggesting heightened perceptual abilities. The Slow Processing Theory [13] points to delays in perceptual and motor processing. However, despite their contributions, these theories often fail to provide a unified account of ASD symptomatology.

The Predictive Coding Theory [14] views the brain as an inferential process, constantly generating internal models to anticipate sensory input and minimizing prediction errors through ongoing updates. Applied to ASD, the Predictive Coding Theory [15, 16] offers a unifying explanation for both social and nonsocial features by suggesting atypicalities in how predictions are formed or updated (see reviews on prediction in ASD [17, 18]).

Within this framework, several hypotheses diverge on which part of the predictive process may be altered in ASD. The *hypervolatility hypothesis* or similarly the *imbalanced hypothesis* suggests an inflexibly high weight of prediction errors, leading to a world perceived as unstable [18, 19, 20, 21]. Conversely, the *slow updating hypothesis* [22, 23, 24] proposes that individuals with ASD may update internal models more slowly, causing persistent prediction errors and rigid behaviors.

These predictive difficulties may underlie challenges in sensory–motor synchronization and interpersonal timing [25, 26, 27], contributing to social and communicative challenges. This raises the possibility that similar difficulties could affect conversational or musical interactions, altering their flow, timing, and mutual alignment.

## 2 Predictive Coding in Conversational and Musical Interactions

### 2.1 Predictive Coding in Conversational Interactions

Conversational interaction is a dynamic and reciprocal process, requiring continuous coordination between interlocutors. According to the predictive coding view, successful conversation relies on the ability to predict both the content and timing of a partner's speech [28, 29, 30], facilitating the seamless turn‐taking that characterizes natural dialogue.

Indeed, turn‐taking is not reactive but anticipatory, participants often planning their response while their partner is speaking. This ability rests on finely tuned mechanisms that allow individuals to anticipate when a turn will end (based on prosodic, semantic, syntactic, and pragmatic cues) and what it will be about, enabling rapid and contextually appropriate responses [31].

We are constantly preactivating linguistic information before it is uttered by our interlocutors [32]. Such preactivations have been shown, for example, in eye‐tracking studies, and rely on semantic [33, 34, 35] or syntactic structure [36]. Garden path sentences [37, 38, 39] provide a well‐known illustration of how these predictions can even mislead listeners toward an incorrect interpretation.

Effective communication also involves alignment occurring across multiple levels of linguistic representation, including lexical, syntactic, semantic, and prosodic levels [40]. The *interactive alignment hypothesis* [41, 42] supports the idea that conversational partners converge on shared mental representations, which in turn reduces processing load and enhances mutual prediction. Alignment, in this sense, is not merely mimicry, but an emergent property of dialogic interaction that optimizes communicative efficiency and supports fast, adaptive responses.

These behavioral and linguistic forms of alignment are increasingly being linked to their neural correlates, where similar patterns of activation are observed across interlocutors during communication [43]. Furthermore, successful coordination in conversation seems to induce upregulated neural activity and involve temporally aligned brain activity, supporting the idea of a dynamic, predictive exchange of information [44, 45, 46].

Together, these findings point to conversation as a multilevel predictive process: from the anticipation of turns and semantic content to the entrainment of speech patterns and neural dynamics. Understanding how predictive coding governs these processes sheds light on the neurocognitive architecture of human communication, and positions dialogue as a rich model system for studying interpersonal coordination more broadly.

### 2.2 Predictive Coding in Musical Processing and Musical Interactions

Throughout this section, we refer to related but distinct constructs: rhythmic processing, rhythmic synchronization, and temporal prediction. Rhythmic processing typically refers to the perception and internal representation of temporal regularities (e.g., is it a march or a waltz?). Rhythmic synchronization involves motor alignment with external rhythmic cues, often studied through tapping or movement paradigms. Temporal prediction refers more broadly to the anticipation of upcoming events in time, whether sensory or motor, and is often conceptualized within computational frameworks such as predictive coding.

Music is a structured yet dynamic phenomenon that engages the brain's predictive capacities in complex, socially embedded ways. Like speech and language, predictive coding explains music perception and production, where the brain continuously updates models to predict sensory input. This framework has been particularly influential in understanding how listeners anticipate and interpret musical structure, rhythm, and harmony.

Recent work has elaborated how predictive coding underlies music processing at multiple levels. Vuust et al. [47] propose that music perception involves an interplay of top‐down expectations and bottom‐up sensory signals, with prediction errors driving learning. Similarly, Koelsch et al. [48] highlight how the hierarchical nature of musical structure maps onto neural mechanisms of predictive processing, suggesting that listeners use prior knowledge to anticipate future musical events across different temporal scales. When we listen to music, we are constantly making predictions about what will happen next (in melody, rhythm, harmony, etc.). Music lets us test and revise these predictions (the *epistemic offering* of music), with a rewarding cycle of uncertainty and resolution. This framework has also been used to explain the feeling of groove, wherein predictions would be mediated by the dorsal pathway via audio–motor coupling of neural activity [49].

The predictive coding framework has also been extended to the domain of musical interactions, such as joint performance or improvisation. In these settings, successful interaction relies on shared predictive models and goals. Keller et al. [50] argue that performers synchronize through mutual prediction and adaptation. This view is echoed by Clayton et al. [51], who propose that rhythmic alignment reflects both sensorimotor coupling and predictions about coperformers' intentions. Wiltshire and Fairhurst's work [52] further supports this by examining predictive alignment in joint musical tasks.

Beyond behavioral coordination, neuroscientific studies reveal neural correlates of predictive coding in musical interactions. In particular, research on interbrain synchrony provides evidence for shared neural dynamics during joint music‐making. Overall, there seems to be a positive correlation between neural synchrony among performers on one side and coordination quality and shared predictive frameworks on the other side [53, 54, 55, 56].

Together, these findings are a promising integration of predictive coding theory with both neuroscience of music and social interaction. Understanding how predictive mechanisms operate not only within but also between individuals during music‐making opens new avenues for exploring the embodied, social, and communicative dimensions of musical experience.

### 2.3 Links Between Conversational and Musical Interactions Under the Prism of Predictive Coding Theories

Recent research suggests that musical interaction can serve as a model for understanding the complexity of human social interactions, with both language and music relying on shared predictive coding mechanisms. In both domains, individuals anticipate what their partner will express (content) and when it will happen (timing), enabling smooth turn‐taking in conversation and coordination in musical performance.

Temporal alignment is a key commonality. Both music and conversation involve continuous prediction of timing, using prosodic, rhythmic, and structural cues to anticipate turn transitions and synchronize responses. Studies, such as those by Jungers et al. [57] and Hadley and Pickering [58], suggest that predictive timing mechanisms operate similarly in both domains. Additionally, research by Wynn et al. [59] found that individuals with better rhythm perception show greater alignment in speech rate during conversations, linking rhythmic acuity to improved conversational quality.

Research on how musical training or rhythmic interventions affect conversational dynamics (or vice versa) is limited. Notably, Robledo et al. [60] showed that brief rhythmic interventions improved conversational flow, suggesting that rhythmic entrainment can enhance communicative coordination. Similarly, studies on clinical populations [61] have explored rhythmic engagement's role in improving turn‐taking like behavior.

Overall, both musical and conversational interactions rely on similar predictive processes, particularly in temporal and content alignment. Musical interaction, with its structured yet improvisational nature, offers a unique lens for studying how predictive coding operates not only within individual brains but also between them, enabling dynamic and adaptive social coordination.

## 3 Speech and Music in ASD Under the Prism of Predictive Coding

### 3.1 Speech Alignment and Turn‐Taking in ASD

**FIGURE 1:**
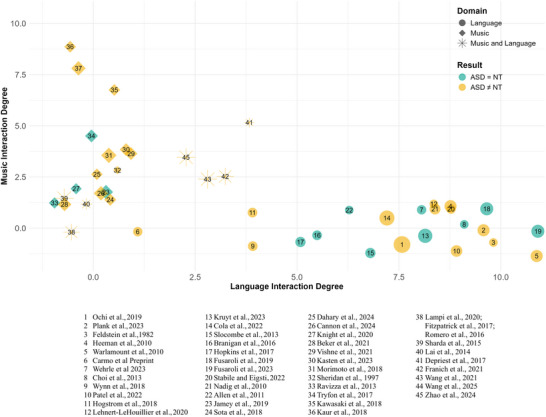
Review of studies assessing music rhythmic abilities and language alignment in autism spectrum disorder (ASD). Each symbol represents one study, plotted as a function of the degree of interaction in the music and language tasks (e.g., describe a picture = 3, free conversation = 10, pitch perception = 0, free music improvisation = 10). Language studies are represented by circles, music studies by diamonds. Studies assessing both are represented by stars. The size of symbols represents the sample size of the ASD group (range: 10–65). The color represents presence (yellow) or absence (turquoise) of differences between ASD and controls.

Voice characteristics in ASD individuals have been the subject of sustained research interest, dating back to the earliest characterizations of the neurodevelopmental profile (see meta‐analysis [62]). Nevertheless, although social interactional challenges are central to ASD, dynamic aspects of interaction, such as turn‐taking and linguistic alignment, have only recently gained attention in ASD language research. Progress in the field remains fragmented due to variability in the developmental stages examined, individual differences, methodological approaches, and the ecological validity of study context. Figure 1 (circles) offers a schematic representation of studies on language alignment in ASD and neurotypical (NT) individuals according to the degree of ecological interaction: this ranges from highly structured, controlled, and not interactive settings (i.e., Language Interaction Degree = 0), to highly ecological and interactive, conversational settings (i.e., Language Interaction Degree = 10).

Linguistic alignment, or the tendency of interlocutors to adapt their language at lexical, syntactic, and phonetic levels, plays a crucial role in dialogue coordination, language processing, and social bonding (the same phenomenon has been referred to with other terminologies including linguistic entrainment, convergence, synchrony, and accommodation (see Refs. [63, 64, 65] for reviews)). Given the variability in communication and language outcome variability across the spectrum [66, 67], studying alignment in ASD individuals offers insights into underlying cognitive processes and has potential implication for intervention, relationship establishment, and quality of life outcomes [68, 69, 70].

The Interactive Alignment framework proposed by Pickering and Garrod [41, 42] posits that perception and production share representations, leading to automatic multilevel alignment that supports predictive processing and smooth conversation, hence communication. Investigating how this mechanism operates—or is altered—in individuals with ASD may offer valuable insights into the nature and the variability of their interactional and language challenges and the underlying cognitive processes involved.

What follows is a brief review of studies on linguistic alignment in ASD, organized according to different levels of linguistic analysis. This selection was informed by targeted PubMed and Google Scholar searches combining terms such as “autism spectrum disorder,” “speech,” “language,” “alignment,” “entrainment,” “convergence,” and “turn‐taking,” alongside manual review of references from key reviews and empirical studies without any age range limitations.

Turn‐taking, an aspect of conversation where prediction is most evident (see section “Predictive coding in conversational interactions”), is often slower in individuals with ASD, marked by longer pauses and silence gaps [71, 72, 73, 74, 75, 76, 77]. To our knowledge, only a few studies show contrasting results. Wehrle et al. [78], using a map task, found that ASD adults showed slower turn‐taking only early in the interaction. After approximately 38 turns (average first‐mismatch time point), their pace aligned with NT participants. This suggests they may need a longer time to adapt to the interlocutor. Similarly, Ochs et al. [79] and Choi and Lee [80] reported intact turn‐taking dynamics in children with ASD, though these studies lacked precise timing data. Wehrle et al. [78] further noted that NT participants delayed turn‐taking more after unpredictable items (mismatches), whereas ASD participants showed less variation (i.e., less shortening for predictable items), indicating lower sensitivity to the degree of predictability of items.

To our knowledge, only two studies have examined syllable rate entrainment in ASD—‐Wynn et al. [81] and Patel et al. [82]—both of which report a lack of alignment in individuals on the ASD, in contrast to NT individuals. Notably, Wynn et al. found an absence of such entrainment not only in ASD adults and ASD children, but also in NT children, suggesting that temporal alignment in conversation may have a developmental trajectory. Consistent with this, Mazzocconi et al. [83] similarly found age‐related differences in laughter mimicry, with only adults showing reliable temporal alignment.

Findings on pitch or prosodic entrainment remain mixed: some studies report preserved prosodic convergence in individuals with ASD (adults [72], adolescents [84], and children [85]), while others find reduced prosodic alignment compared to NT individuals (adults [71, 82] and children [86]).

In Hogstrom et al. [84], a difference between groups was observed only at the level of phonic realization of the phoneme(s) where NT individuals converged on its duration and ASD individuals did not. The degree of convergence was negatively associated with the level of autistic traits and atypical sensory profiles, suggesting that social and sensory processing differences may impact phonetic alignment. This study, though, was based on monologic recordings of sentences read before and after a collaborative interaction, therefore calling into question the generalizability of the results.

Studies on intensity alignment are similarly inconsistent. Ochi et al. [71] reported no alignment in adults with ASD while Plank et al. [72] reported increased alignment compared to NT participants. Cola et al. [87] observed alignment in NT adults, but not in ASD adults for talkativeness and approach to the task (bored/interested).

In contrast, lexical and syntactic alignment appear largely preserved in ASD. Most studies report no significant differences between ASD and NT individuals, either in adults [82, 88] and children [89, 90, 91, 92, 93], while only two studies in children and adolescents showed reduced lexical alignment [94, 95]. Interestingly, Fusaroli et al. [91] show that lexical and semantic alignment from the child increases longitudinally (highlighting again the developmental aspects of alignment), but such an increase was slower for children with ASD.

The relative consistency in syntactic alignment suggests similar linguistic representations in ASD at this level of processing, across both adults [82, 88] and children [90, 91, 92, 93, 96]. Kruyt and Beňuš [97] propose that differences in acoustics and prosodic alignment may arise from these features serving more paralinguistic functions. While we do emphasize that prosody also carries linguistic and pragmatic functions, it seems plausible to suggest that individuals with ASD have fully preserved linguistic representation alignment at some levels (i.e., lexicon and syntax), but reduced alignment on more temporally dynamic features, such as prosody, intensity, and syllable rate.

Overall, the state of the art offers a scattered picture reporting often inconsistent findings due to the wide variability in experimental designs, ecological validity, and methods of analysis. In particular, we highlight that, with the exception of Wehrle et al. [78], few studies consider alignment over the time–course of interaction.

We also stress the need for studies considering both matched and mixed neurotype dyads (as done in Wehrle et al. [78]). Recent evidence suggests the social difficulties experienced by individuals with ASD may stem from neurotype mismatches rather than intrinsic deficits [98, 99]. This is especially relevant when investigating alignment since it is highly modulated by the familiarity or perceived closeness between interactants [100]. Furthermore, one should consider that atypical alignment by individuals with ASD may influence their partners’ behavior. Difficulties in predictions from autistic individuals, and eventual reduced alignment, could in turn lead to difficulties in prediction from the NT interactant resulting in atypical turn‐taking or alignment patterns. So far, this has been investigated primarily in the context of development [101, 102].

## 4 Rhythmic Processing and Rhythmic Synchronization Abilities in ASD

### 4.1 Rhythmic Processing in ASD

While pitch processing in ASD has been widely studied and generally found to be preserved—or even enhanced—rhythmic processing has received comparatively less attention. Yet, the ability to perceive and anticipate temporal structures in music plays a critical role in predictive and multisensory integration processes, which are often hypothesized to function differently in autism.

Figure 1 (diamonds) offers a schematic representation of studies on rhythmic skills in ASD and NT individuals according to the degree of ecological interaction. The study selection was informed by targeted PubMed and Google Scholar searches combining terms such as “autism spectrum disorder,” “rhythm,” “beat perception,” “entrainment,” and “predictive coding,” supplemented by manual review of references from recent music cognition and neurodevelopmental studies.

**FIGURE 2:**
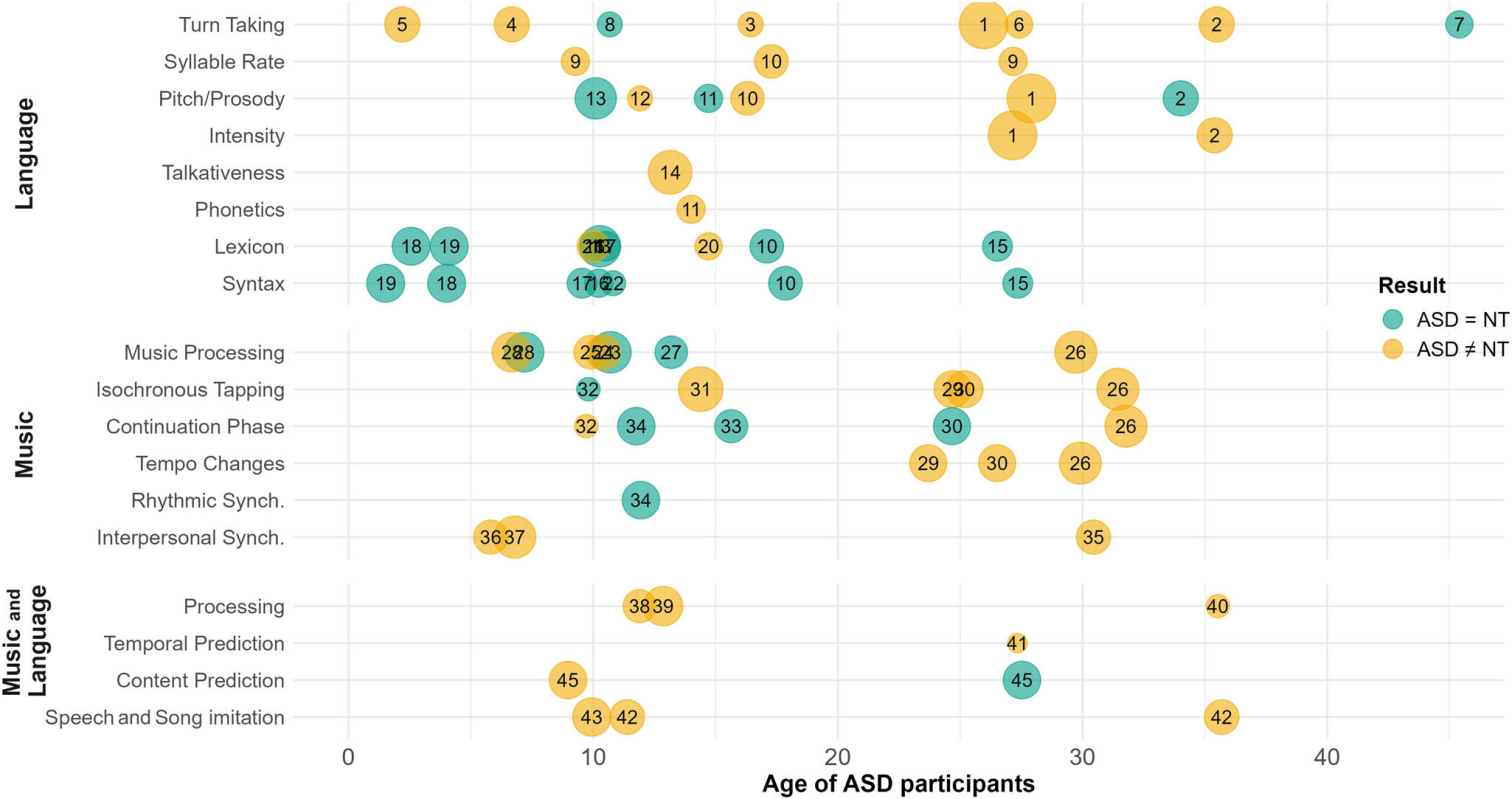
Review of studies assessing language alignment, music rhythmic abilities, and music and language in autism spectrum disorder (ASD). Each symbol represents one study, plotted as a function of the age of participants and the precise measure assessed. The size of symbols represents the sample size of the ASD group (range: 10–65). The color represents presence (yellow) or absence (turquoise) of differences between ASD and controls.

Numerous studies have consistently shown preserved or enhanced pitch and melodic processing in individuals with ASD [103, 104, 105, 106] (but see Refs. [107, 108] for evidence of heterogeneity in the literature, potentially moderated by individual cognitive differences). However, studies focusing more specifically on rhythmic processing of music in ASD remain relatively scarce (Figure 2). Two studies used similar batteries (MBEA‐s and MBEMA), where participants judge whether two musical sequences are similar or different in pitch or rhythm [106, 109]. Both found preserved rhythmic discrimination abilities in children with ASD, although Sota et al. [109] surprisingly found differences in the melodic subtest. Importantly, these tasks may rely more on short‐term memory than rhythmic processing or temporal prediction, limiting conclusions about beat perception and related predictive abilities.

To address this, Dahary et al. [110] implemented an adaptation of the Beat Alignment Test (BAT), where children with ASD and NT children identified whether superimposed beeps on music aligned with the beat. They found that while children with ASD were equally accurate at detecting on‐beat alignments, they were less precise at detecting beat misalignment (off beat). This asymmetry is interesting in light of the greater multisensory integration required in the off‐beat condition. Indeed, while rhythmic perception could be preserved overall in ASD, performance could be disrupted when tasks require integrating variable or complex sensory input. This aligns with the *hypervolatility hypothesis* of autism, which proposes that individuals with ASD overestimate the unpredictability of the environment. However, it is worth noting that task performance was below the statistical chance threshold of 70% across both groups and that NT participants had more musical training, potentially confounding results.

In the same vein, Cannon et al. [111] used a metronome‐based task where adults judged whether the last tone in a sequence was early or late. While there were no differences in the point of subjective isochrony, participants with ASD exhibited higher perceptual timing noise—although this effect may have been driven by outliers. Together, these findings suggest that basic rhythmic processing is broadly preserved in ASD, although subtle atypicalities may emerge under conditions of increased complexity or sensory load.

Efforts to explicitly link rhythmic processing with predictive coding theories of autism remain limited. Knight et al. [112], using EEG, examined neural markers of prediction error in response to auditory rhythms of varying complexity. Contrary to expectations, they found no detectable differences in autistic participants compared to NT individuals, even under increased rhythmic complexity. These results challenge the idea of a global atypicality in temporal prediction in ASD, at least in contexts involving relatively low ecological demands. Lisøy et al. [113] approached this question from a different angle, examining individual differences in the “musical sweet spot”—the peak of the inverted U‐shaped curve between music complexity and liking. Based on the hypothesis that individuals with higher autistic traits may prefer more predictable, less complex music due to heightened sensitivity to uncertainty, they expected shifts in the sweet spot. While some variability was observed, no clear link with autistic traits emerged. Future studies could benefit from testing these hypotheses in clinically diagnosed populations, where stronger predictive effects might appear, rather than relying solely on self‐report measures in the general population. While these studies focus on auditory rhythms, evidence from visual cueing paradigms also points to reduced neural entrainment despite preserved behavioral prediction in ASD [114], suggesting that atypical rhythmic entrainment may not be restricted to the auditory domain.

### 4.2 Auditory–Rhythmic Synchronization With Rhythmic Patterns in Children With ASD

To our knowledge, only one study to date has investigated synchronization to rhythmic patterns in ASD. Tryfon et al. [115], asked children with ASD and NT children to tap along to rhythmic patterns of varying metrical complexity. Results showed no significant group differences, with both groups improving with age and performing worse as metrical complexity increased. Although these results appear consistent with findings from studies on rhythmic processing in ASD, it seems crucial to replicate the results observed in this single study and expand these observations using tempo variations between or within trials, which would better capture the kind of volatility and adaptability challenges that may reveal differences in ASD.

### 4.3 Auditory–Rhythmic Synchronization During Metronome‐Based Tapping Tasks in ASD

In contrast to pattern‐based synchronization, metronome‐based tapping tasks have been more widely used. Although they do not involve musical rhythm per se, these tasks are highly informative for studying predictive abilities, as they require rapid integration of sensory feedback and error correction (i.e., internal model updating).

Several recent studies [23, 24, 111] used the same computational model to dissect the sources of timing variability, error correction, timekeeper noise, and motor noise. While all studies reported an increased variability of asynchrony during isochronous tapping in the autistic groups, they also found significant differences in the main outcomes of the computational model. Indeed, Vishne et al. [23] and Kasten et al. [24] found no intergroup differences in timekeeper noise (in line with Refs. [116, 117, 118]), or in motor noise. However, they did observe a significantly reduced phase correction in the autistic group, meaning they took longer to adjust for timing errors, consistent with the *slow updating hypothesis* of autism. By contrast, Cannon et al. [111] reported increased temporal noise in the ASD group but no differences in phase correction or motor noise. These discrepancies may stem from methodological variations, such as the interonset interval used (700 ms vs. 500 ms in the other studies) or group imbalances in musical training, which Cannon attempted to address through group balancing.

Other studies have also reported increased variability in tapping performances, both in adolescents [119] and adults [120]. Only Sheridan and McAuley [121], with a small sample size (*N* = 17) reported no difference. Overall, the trend across studies supports increased variability and reduced precision in rhythmic motor synchronization in autism.

### 4.4 Continuation Phase and Adaptation to Tempo Changes

Variants of this task involve stopping the metronome and asking participants to continue tapping at tempo. This continuation phase allows the study of tempo‐keeping by analyzing the variability of participant's intertap intervals (ITIs). Results here are mixed: while Cannon et al. [111], and Sheridan and McAuley [121] reported greater continuation noise in ASD, others found no differences [24, 115, 122]. Notably, Kasten et al. [24] found that ASD participants exhibited a greater reduction of ITI variability from the synchronization phase to the continuation phase, suggesting that they may benefit from reduced external volatility, a finding consistent with the *hypervolatility hypothesis* (less multisensory integration could lead to better performance). Sudden tempo shifts can also be introduced to test adaptability. In Vishne et al. [23] random tempo changes were inserted throughout the trial and autistic participants showed reduced adjustments to these changes, indicating inflexible internal model updating. Kasten et al. [24], used a different approach, with a single acceleration or deceleration per trial, and found a significant group difference only in the acceleration condition. The reduced volatility of this design may explain the lack of broader group differences. Finally, Cannon et al. [111] also found reduced error correction in response to tempo perturbations in the balanced autistic group.

### 4.5 Interpersonal Auditory–Rhythmic Synchronization in ASD

Finally, studies investigating interpersonal auditory–rhythmic synchronization abilities in autism, that is, coordination with another individual in time, are even rarer than those examining individual rhythmic skills.

Kawasaki et al. [123] examined antiphase synchronization abilities in adults with ASD. Participants tapped a key back and forth at a steady tempo with either a constant virtual partner, a variable virtual partner and a NT human partner. Results showed preserved antiphase synchronization abilities with the constant virtual partner but impaired performance with both the variable virtual and human partners. These findings raise the interesting idea that synchronization difficulties in autism may be more closely related to stimulus variability than to a simple distinction between social and nonsocial stimuli.

Other studies have investigated interpersonal synchronization in children with ASD using rhythmic cueing. For instance, Kaur et al. [124] highlighted impaired interpersonal rhythmic synchronization in children with ASD while they were either clapping, marching, marching and clapping, or drumming with the experimenter. Children with ASD spent less time in synchrony with the adult partner compared to NT peers. Conversely, Yoo and Kim [125], raised the possibility that rhythmic cueing may facilitate interpersonal synchronization: they observed reduced asynchronies during a drumming task with an adult partner when rhythmic cues were present, compared to a no‐cue condition. However, this study involved a small sample (10 participants with ASD) and did not include any group comparison, limiting the strength of its conclusion.

In another series of studies [126, 127, 128] (all based on the same dataset), researchers found reduced rhythmic coherence during hand clapping and drumming tasks in autistic children compared to controls. However, a dissociation seems to emerge between imitation and synchronization phases. For instance, the between‐group differences in coherence seem greater during simultaneous synchronization than during sequential imitation [127]. Similarly, Romero et al. [128] reported equivalent occurrences of antiphase coordination across groups. These findings may be relevant to the *hypervolatility hypothesis*, as they suggest that individuals with ASD may perform better when allowed to respond after a delay, rather than synchronously. This would reduce the need to continuously predict and adapt in real time.

Overall, higher temporal variability during interpersonal auditory–rhythmic synchronization seems to be a recurrent finding in autism. However, the underlying mechanisms are poorly understood. Future research is needed to determine whether they are primarily driven by social factors or by the increased unpredictability of human partners, which may trigger the same predictive adaptation challenges observed in nonsocial, motor‐based tasks [23].

The current body of literature provides a nuanced view of rhythmic abilities in ASD, suggesting a general preservation of basic rhythmic processing but a more complex and heterogeneous picture when it comes to rhythmic synchronization—especially in contexts that require real‐time adaptation or interpersonal coordination.

Tasks involving simple beat perception or isochronous tapping already show more variable performance in ASD. More demanding conditions, such as off‐beat detection, tempo changes, or social coordination, confirm increased variability or reduced adaptation. These findings support predictive coding accounts of autism, particularly the *hypervolatility hypothesis*, which posits heightened sensitivity to environmental unpredictability.

Interpersonal studies further suggest that stimulus variability, rather than sociality per se, may underlie synchronization challenges. Notably, individuals with ASD often perform better in predictable or sequential interactions than in simultaneous or highly variable ones—highlighting the importance of temporal predictability over social context alone.

Overall, these results underscore the value of rhythm as a lens on predictive mechanisms in ASD, and call for more ecologically valid, computationally informed studies that bridge simple motor tasks and real‐world social timing.

## 5 Links Between Music and Speech in ASD Under the Prism of Predictive Coding Theories

Figure 1 provides an overview of studies investigating music and/or speech in ASD. It shows that studies addressing both domains (star symbols) remain limited, particularly in ecologically valid interactive contexts, highlighting a need for more integrative research frameworks. Some have reported preserved music processing alongside specific atypicalities in speech processing in children and adults with ASD [129, 130, 131], suggesting distinct neural mechanisms but potential interconnections. Others also found a positive correlation between phonological awareness and beat perception in children with ASD, further supporting the idea of shared underlying mechanisms [132]. Similarly, studies on vocal imitation in ASD have reported parallel difficulties across speech and song, particularly in matching pitch and duration features, suggesting partly overlapping processes [133, 134]. To our knowledge, only two studies have made a direct link between speech, music, and predictive coding in ASD.

Franich et al. [120] found that timing variability was greater and correlated across both speech‐based metronome repetition and drum tapping tasks in autistic individuals, suggesting that shared temporal predictive mechanisms may be disrupted. This finding supports the hypothesis that predictive timing, rather than domain‐specific processes, could underlie communicative challenges in ASD.

In contrast, Zhao et al. [135] used both production and perception tasks to assess predictive abilities in music and language. Participants either completed unfinished melodies or sentences, or judged the expectedness of the completions. Their results showed that performance on musical and linguistic prediction in autism is not necessarily impaired, but it rather is largely dependent on individual differences in musical, linguistic, and cognitive abilities. However, it is worth noting that this study focused on content prediction rather than temporal prediction. This contrast between temporal versus content predictions echoes the “slow update hypothesis,” which posits that autism involves delayed updating of predictive models, particularly in timing. This distinction underscores the need for studies that assess both content and temporal prediction, ideally in ecological settings.

Despite the lack of studies linking speech and music in ASD via the predictive coding framework, the relevance of predictive models is supported by recent works on developmental speech and language disorders [136] and on neurodevelopmental disorders more broadly [137]. In these studies, the authors highlight the connections between these disorders, which often share common comorbidities and in which atypicalities in rhythmic and timing abilities appear to be a ubiquitous feature.

Overall, as can be seen in the relatively scarce number of studies assessing both language and music abilities in ASD (Figure 2), there is a need for increasing these empirical connections, in particular between speech alignment and musical/rhythmic synchronization abilities. This connection could open promising clinical perspectives, although more experimental evidence is needed to establish whether enhancing rhythmic synchronization abilities causally impacts communication outcomes in ASD [138, 139, 140, 141].

There is growing recognition among clinicians of the positive effect of music on social communication skills in autism, and it has been increasingly explored and applied in clinical practice (e.g., through music therapy). However, the corresponding studies often include rather small samples (median sample size = 24 participants [142]) and/or rely on limited outcome measures. Most studies rely solely on standardized scales, except for a few that explored additional outcomes, such as brain connectivity [143], executive functioning [144], annotated visual data, [145] or word count and verbalization duration [146].

Across music and speech, evidence shows that while basic perceptual and motor abilities can be impaired in ASD, difficulties are exacerbated during real‐time adaptation, especially under conditions of increased temporal variability or multisensory complexity. These findings support predictive coding accounts of autism, particularly the “hypervolatility” and “slow updating” hypotheses, which propose heightened sensitivity to unpredictability and delayed internal model updating.

Despite this, relatively few studies have explored the connection between musical and linguistic temporal processing in ASD. The overlap in temporal demands across these domains points to shared predictive mechanisms that could underlie challenges in both rhythmic synchronization and conversational turn‐taking. However, individual differences and task‐specific factors also play a role, suggesting a need for more nuanced investigation.

This research has important clinical implications. Music therapy is already widely used in ASD interventions, yet little is known about how rhythmic training might affect speech timing or social coordination. Bridging these areas could lead to more targeted and effective therapies. Future studies should adopt ecologically valid, interactive designs to explore how improvements in musical timing might support communicative skills. Rhythm may be more than a diagnostic clue—it could become a powerful tool for improving social interaction and adaptability in autism.

## 6 Conclusion and Future Directions

In this review, we examined how the predictive coding framework sheds light on the shared temporal dynamics of music and language processing in individuals with ASD. Across the domains of conversational and musical interaction, we found evidence that temporal prediction, in particular in real‐time, interactive contexts, is often atypical in ASD. Challenges are particularly evident when tasks require rapid adaptation, integration of multisensory input, or coordination with a partner. These patterns are consistent with predictive coding accounts of ASD, particularly the “hypervolatility” and “slow updating” hypotheses.

An emerging observation from the reviewed literature is that music and language share common demands for temporal prediction, turn‐taking, and alignment. Yet, surprisingly few studies have directly linked musical and linguistic temporal processing in autism, and even fewer have done so within ecologically valid, interactive settings. This gap limits our ability to determine whether difficulties in one domain (e.g., rhythmic entrainment) correspond to challenges in another (e.g., conversational timing).

We acknowledge that this review was conducted as a narrative synthesis rather than a systematic review. While our aim was to allow conceptual integration across diverse literatures, our approach does not follow a predefined protocol with exhaustive search criteria, and may have overlooked some relevant studies, especially in areas with fast‐evolving or interdisciplinary findings. Thus, a full systematic or meta‐analytic review remains an important future step for mapping this growing field.

Several other limitations in the existing literature also warrant attention. Many studies rely on simplified, decontextualized tasks, making it difficult to generalize findings to real‐world social interactions. Others fail to account for individual variability in cognitive abilities, musical training, or neurotype pairing during interaction. Moreover, relatively few studies examine the developmental trajectories of predictive abilities or test both domains (music and speech) within the same participants.

Future research would benefit from integrative designs that assess predictive timing in both language and music domains, ideally using interactive paradigms, longitudinal samples, and computational modeling. Studies should explore whether musical training or rhythmic intervention can enhance not just motor timing, but also communicative coordination. Particular attention should be paid to mixed–neurotype interactions, which may provide deeper insights into the role of interpersonal prediction mismatches.

## Author Contributions

Maude Denis and Chiara Mazzocconi contributed to the conceptualization, methodology, investigation, data curation, visualization, writing original draft, reviewing and editing previous versions of the manuscript; David Da Fonseca contributed to the reviewing and editing of previous versions of the manuscript, supervision; Daniele Schön contributed to conceptualization, methodology, investigation, data curation, writing original draft, reviewing and editing previous versions of the manuscript, supervision, project administration, and funding acquisition. All the authors have read and approved the last version of the manuscript.

## Conflicts of Interest

The authors declare no conflicts of interest.

## Peer Review

The peer review history for this article is available at: https://publons.com/publon/10.1111/nyas.70084.

## Data Availability

Data sharing not applicable to this article as no datasets were generated or analyzed during the current study.
